# Supplementary material for: Immunization with centrin-Deficient Leishmania braziliensis Does Not Protect against Homologous Challenge
Source: Vaccines (Basel). 2024 Mar 15;12(3):310. doi: 10.3390/vaccines12030310 (PMC10974367; doi:10.3390/vaccines12030310)
Supplement: Supplementary file 1 [file vaccines-12-00310-s001.zip › vaccines-2886024-supplementary.pdf]

Supplementary Material

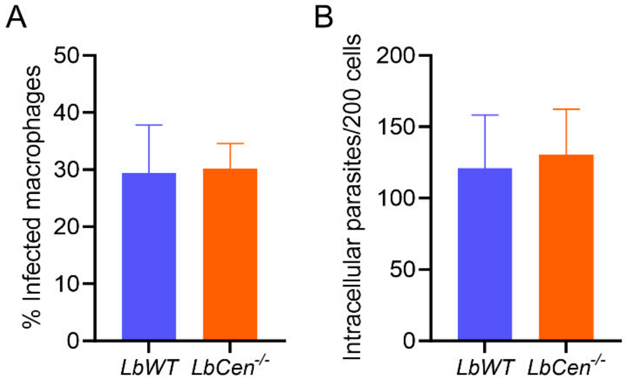

Supplemental Figure S1. Uptake of *LbCen*$^{-/-}$ and *LbWT*. Bone marrow-derived macrophages (BMDM) were co-culutured with *LbWT* or *LbCen-/-* promastigotes. After 6h, cells were washed and stained with H&E. The percentage of infection (A) and the number of amastigotes per 200 macrophages (B) was determined by optical microscopy. Results are presented as means ± SEM and are pooled from two independent experiments, each performed in quadruplicate.

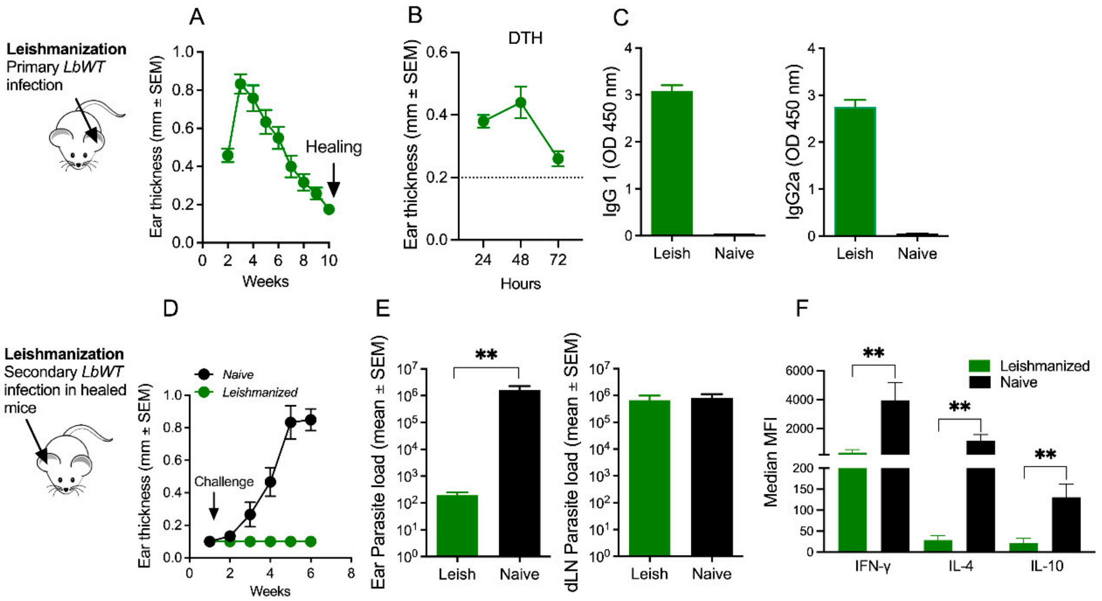

Supplemental Figure S2. A primary infection with *L. braziliensis* confers immunity against subsequent challenge. BALB/c mice were inoculated with *LbWT* promastigotes and (A) lesion development was measured weekly. (B) DTH response to SLA and anti-SLA (C) IgG1 and (D) IgG2a, determined by ELISA, were evaluated ten weeks after the primary infection. (D) Healed (leishmanized) mice were challenged with *LbWT* promastigotes and lesion development was measured weekly. Controls consisted of age-matched naïve mice. (E) Parasite load was evaluated five weeks after challenge, by limiting dilution. (F) Cytokine production was evaluated five weeks after challenge, in dLNs cell in response to SLA . Results are expressed as means ± SEM and are from one representative experiment. **p<0.01.
